# Biomimetic bioreactor for potentiated uricase replacement therapy in hyperuricemia and gout

**DOI:** 10.3389/fbioe.2024.1520663

**Published:** 2025-01-07

**Authors:** Bin Yang, Guihu Luo, Tailei Nie, Zhenglan Ban, Quanxin Ning, Jialin Zhang, Xiangru Liu, Yanhua Lin, Xiaochun Xie, Qianyun Chen, Han Zhong, Ying Huang, Pan Liao, Yan Liu, Chenyang Guo, Chuanxu Cheng, Erwei Sun

**Affiliations:** ^1^ Department of Rheumatology and Immunology, The Third Affiliated Hospital of Southern Medical University, Institute of Clinical Immunology, Academy of Orthopedics, Guangzhou, Guangdong, China; ^2^ Department of Rheumatology and Immunology, Shunde Hospital of Southern Medical University (The First People’s Hospital of Shunde), Foshan, China; ^3^ Department of Pharmacy, Affiliated Hospital of Yangzhou University, Yangzhou University, Yangzhou, Jiangsu, China; ^4^ National Engineering Research Center for Tissue Restoration and Reconstruction, South China University of Technology, Guangzhou, Guangdong, China; ^5^ Xingtan Hospital Affiliated of Southern Medical University Shunde Hospital, Foshan, China; ^6^ School of Medicine, South China University of Technology, Guangzhou, Guangdong, China; ^7^ Department of Rheumatology and Immunology, Hunan University of Medicine General Hospital, HuaiHua, China

**Keywords:** uricase, red blood cell coating, biomimetic bioreactor, selenium-based nanoscavenger, hyperuricemia, gout

## Abstract

**Introduction:**

Uricase replacement therapy is a promising approach for managing hyperuricemia and gout but is hindered by challenges such as short blood circulation time, reduced catalytic activity, and excessive hydrogen peroxide (H_2_O_2_) production. These limitations necessitate innovative strategies to enhance therapeutic efficacy and safety.

**Methods:**

We designed and synthesized RBC@SeMSN@Uri, a red blood cell-coated biomimetic self-cascade bioreactor, which encapsulates uricase (Uri) and a selenium-based nano-scavenger (SeMSN) within RBC membranes. This design aims to reduce immunogenicity, extend systemic circulation, and maintain enzymatic activity. *In vitro* assays were conducted to evaluate biocompatibility, anti-inflammatory effects, and oxidative stress protection. *In vivo* experiments in hyperuricemia and gout models assessed therapeutic efficacy, biodistribution, and biosafety.

**Results:**

RBC@SeMSN@Uri effectively degraded uric acid (UA) into allantoin and converted H_2_O_2_ into water, preventing oxidative damage and inflammation. *In vitro* assays demonstrated excellent biocompatibility and reduced H_2_O_2_-induced inflammatory responses compared to free uricase. *In vivo*, the bioreactor prolonged circulation time, significantly reduced uric acid levels, alleviated kidney damage, and mitigated symptoms of hyperuricemia and gout. It also targeted inflamed joints, reducing swelling and inflammation in gouty arthritis models.

**Discussion:**

This study presents RBC@SeMSN@Uri as a novel biomimetic strategy for enzyme replacement therapy in hyperuricemia and gout. By integrating uricase and selenium-based nano-scavenger within RBC membranes, the bioreactor addresses key limitations of traditional therapies, offering enhanced stability, reduced immunogenicity, and superior therapeutic efficacy. This platform holds potential for broader applications in protein or antibody delivery for enzyme replacement therapies in other diseases.

## 1 Introduction

Gout, a prevalent form of inflammatory arthritis affecting nearly 4% of adults (Dalbeth et al., 2021), is caused by hyperuricemia (Dalbeth et al., 2016)—elevated uric acid (UA) levels in the blood. Hyperuricemia is linked to various conditions, including metabolic syndrome and cardiovascular diseases, and promotes monosodium urate crystal deposition in joints, leading to acute and chronic inflammation (Dalbeth et al., 2019). Clinical management of hyperuricemia and gout primarily focuses on reducing UA production and enhancing its excretion (Du et al., 2024; Garay et al., 2012). Uricase, which converts UA to the more soluble allantoin and hydrogen peroxide (H_2_O_2_), is effective in lowering plasma UA levels (Schlesinger et al., 2023). However, its clinical application is hindered by enzyme instability, reduced catalytic activity, and excessive H_2_O_2_ production, which can cause inflammation and other adverse side effects (Lin et al., 2022; Fini and Stenmark, 2019; Hu et al., 2024; Liu et al., 2016; Ming et al., 2021; Tang et al., 2024; Xi et al., 2021; Xi et al., 2024; Zhuang et al., 2020). Therefore, to improve the therapeutic effectiveness of uricase-based treatments in hyperuricemia and related conditions, it is crucial to enhance the enzyme stability, reduce immunogenicity, and ensure rapid elimination of H_2_O_2_ to prevent inflammatory side effects.

Significant efforts have been made over the decades to develop uricase for treating hyperuricemia and gout (Du et al., 2024; Garay et al., 2012; Schlesinger et al., 2023). Although PEGylation is widely used to extend the circulation time of uricase and reduce its immunogenicity, many patients still experience rapid uric acid (UA) accumulation due to quick enzyme clearance (Du et al., 2024). To address these limitations, innovative drug delivery systems have been explored (Ban et al., 2024; Ewii et al., 2024; Li et al., 2024; Liu et al., 2016; Zhang et al., 2018; Zhuang et al., 2020), particularly utilizing red blood cells (RBCs) as carriers (Ban et al., 2024; Li et al., 2024; Zhuang et al., 2020). RBCs offer prolonged circulation, biocompatibility, low immunogenicity, and flexibility. With advancements in nanotechnology, RBC membranes can now be camouflaged onto nanoparticles, preserving their protein repertoire and further enhancing drug delivery with extended circulation and reduced immunogenicity (Hu et al., 2011; Feng et al., 2024; Guo et al., 2020; He et al., 2023; Jiang et al., 2021; Nguyen et al., 2023; Wang et al., 2017; Xie et al., 2019; Yu et al., 2023; Zhao et al., 2022). This RBC membrane-based biomimetic approach shows promise in improving the stability and efficacy of uricase treatments, paving the way for more effective therapies for hyperuricemia and related conditions.

To address rapid H_2_O_2_ elimination, we focus on selenium-based H_2_O_2_ nano-scavenger (Bisht et al., 2022; Hosnedlova et al., 2018). Selenium plays a crucial role in maintaining redox homeostasis and eliminating reactive oxygen species (ROS) *in vivo* (Ghosh et al., 2018; Hosnedlova et al., 2018; Skalickova et al., 2017; Wu et al., 2021; Yoshida et al., 2011), thanks to its biocompatibility and strong hydroxyl radical scavenging ability. As a promising hydrogen peroxide (H_2_O_2_) scavenger, selenium compounds have demonstrated potent ROS-scavenging properties (Cheng et al., 2024; Wei et al., 2024). However, their therapeutic application is often constrained by selenium’s narrow therapeutic window (Bisht et al., 2022). Recent advances in nanotechnology have led to the development of selenium nanoparticles (SeNPs) (Bisht et al., 2022; Chen et al., 2021; Huang et al., 2023; Li et al., 2017; Shao et al., 2018; Shao et al., 2020; Shi et al., 2022; Skalickova et al., 2017; Xu et al., 2023), which offer a safer and more effective alternative to traditional selenium compounds. With unique physicochemical properties, SeNPs exhibit improved bioavailability and reduced toxicity, allowing for enhanced *in vivo* tolerance and targeted delivery to specific tissues or cellular environments. This inspired us to develop a selenium-based nano-scavenger (SeMSN) to leverage selenium’s antioxidant capabilities while avoiding its toxicity. While SeNPs have rarely been explored for gout treatment, utilizing Se-based H_2_O_2_ nano-scavengers presents a promising approach for enhancing H_2_O_2_ scavenging, thereby offering significant benefits for managing gout and related conditions.

**FIGURE 1:**
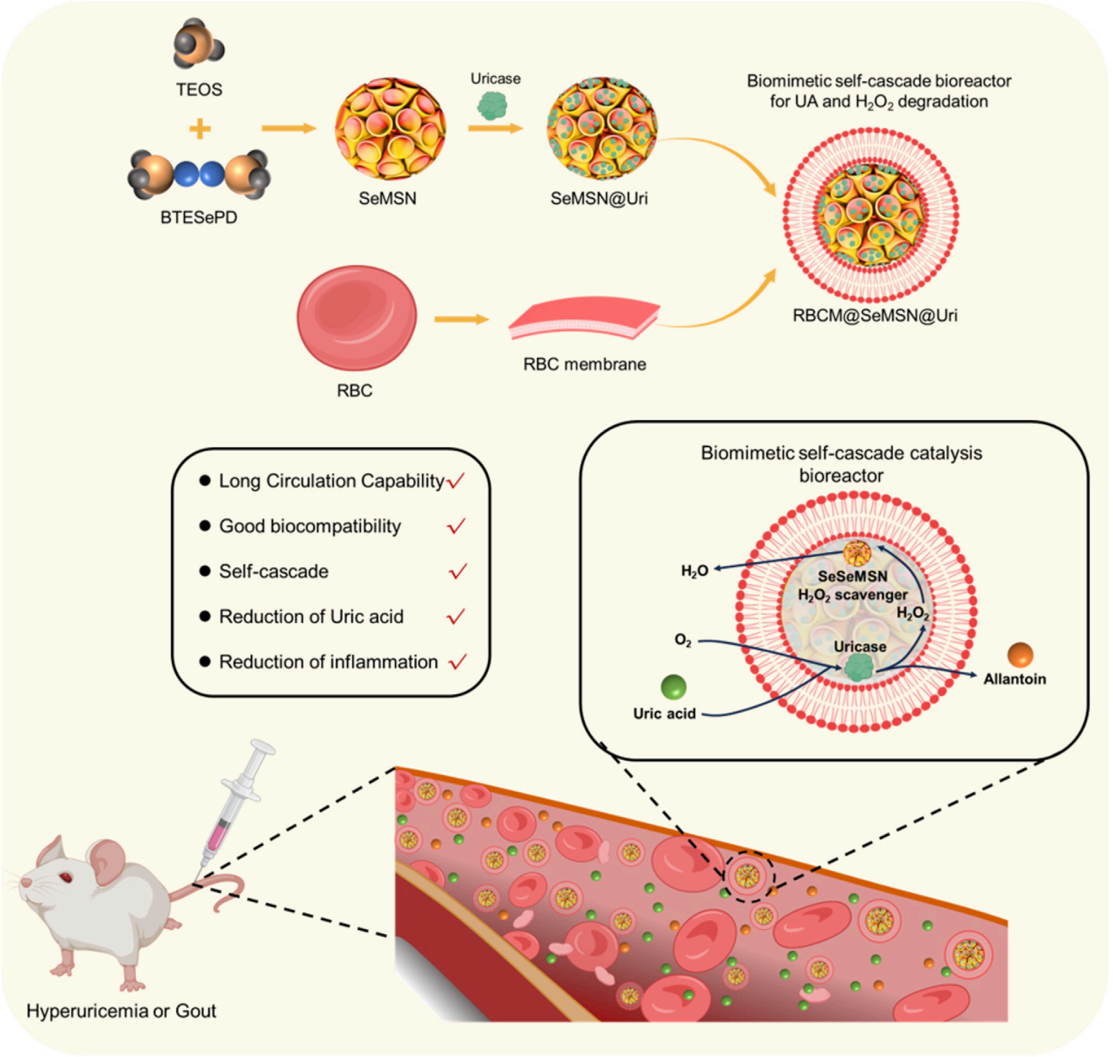
Schematic illustration of the design and synthesis of an RBC-coated biomimetic self-cascade catalysis bioreactor (RBC@SeMSN@Uri) to extend systemic circulation, enhance cell viability, preserve uricase activity, and reduce immunogenicity for treating hyperuricemia and gout.

Here, we designed and synthesized a red blood cell (RBC)-coated biomimetic self-cascade bioreactor as a novel biomimetic strategy for enzyme replacement therapy in hyperuricemia and gout (Figure 1). By encapsulating Uricase (Uri) and selenium-based nano-scavenger (SeMSN) within RBC membranes, we developed the RBC@SeMSN@Uri bioreactor. This design reduces recognition by the reticuloendothelial system (RES), extending systemic circulation, maintaining high cell viability, and preserving enzymatic activity and reduce the immunogenicity of Uricase. The biomimetic bioreactor system effectively degrades uric acid into allantoin and H_2_O_2_, while the selenium-based nano-scavenger further convert toxic intermediates into H_2_O, preventing oxidative damage and reducing H_2_O_2_-induced inflammation. These advantages enable RBC@SeMSN@Uri to significantly alleviate symptoms of persistent hyperuricemia and gout *in vivo* with minimal biosafety concerns. Our study presents the RBC-coated bioreactor as a promising platform for uricase delivery, potentially adaptable for protein or antibody delivery to treat various diseases requiring enzyme replacement therapy.

## 2 Materials and methods

### 2.1 Preparation of RBC@SeMSN@Uri

To prepare Uricase-loaded SeMSN, 800 μg of SeMSN was suspended in 1 mL of deionized water with 800 μg of uricase and stirred at 4°C overnight. The mixture was then centrifuged at 8,000 rpm for 10 min and washed twice with deionized water. The supernatant was collected to measure uricase concentration using BCA Protein Assay Kits. The drug loading content (LC%) was calculated using the following Equation 1:

$$\text{Drug loading content }\left(\%\right)=\frac{w1-w2}{w3+\left(w1-w2\right)}\times 100\%$$ (1)

where W1 is the initial weight of uricase that was added to the SeMSN suspension and W2 is the weight of uricase in supernatant, while W3 is the weight of SeMSN.

For the preparation of RBC@SeMSN@Uri, SeMSN@Uri were combined with a preobtained erythrocyte membrane vesicle solution and cocultured for 30 min under gentle shaking at 4°C, followed by sonication for 1 min. The resulting RBC@SeMSN@Uri were then carefully centrifuged to remove excess membrane components from the supernatant.

### 2.2 Characterization of RBC@SeMSN@Uri

The morphologies of the MSN, MSN@Uri, RBC@MSN@Uri, SeMSN, SeMSN@Uri and RBC@SeMSN@Uri were characterized using a JEM-2100F transmission electron microscope (TEM, JEOL, Ltd., Japan) and a scanning electron microscope (SEM, FEI Quanta 200F). Particle size (diameter, nm), polydispersity, and surface charge (zeta potential, mV) were measured using dynamic light scattering (DLS) on a Zetasizer Nano ZS (model ZEN3600 from Malvern Instruments).

### 2.3 Bone marrow-derived macrophage (BMDM) isolation and culture

BMDMs were derived from the bone marrow, which was isolated from male C57Bl/6 mice and cultured in DMEM (HyClone) supplemented with 1% penicillin/streptomycin (Invitrogen), 10 ng/mL M-CSF (Peprotech) and 10% FBS (Invitrogen).

### 2.4 Cell culture

L-929 cells, RAW 264.7 cells, HUVECs and BMDM were cultured in Dulbecco’s modified Eagle medium (DMEM) at 37°C with 5% CO_2_ in a humidified atmosphere. The medium included 5% fetal bovine serum (FBS) and 1% penicillin-streptomycin.

### 2.5 Hemolysis assay

The mice were housed under SPF conditions with a 12-h dark/light cycle. Whole blood collected from mature mice was washed with 1 × PBS to obtain pure erythrocytes. A 400 μL aliquot of 4% RBCs was mixed with 400 μL of SeMSN or RBC@SeMSN@Uri at various concentrations (12.5, 25, 50, 100, and 200 μg·mL⁻^1^) and incubated at 37°C for 24 h. For controls, the same amount of RBCs was dispersed in saline (positive control) and in water containing 1% Triton (negative control). The mixtures were centrifuged at 500 g at 4°C for 5 min. Hemolysis percentages were calculated using Equation 2:

$$Hemolysis=\frac{{OD}_{sam}-{OD}_{neg}}{{OD}_{pos}-{OD}_{neg}}\times 100\%$$ (2)

### 2.6 H_2_O_2_-induced cellular oxidative damage assay

L-929 cells, RAW 264.7 cells, and HUVECs were used to assess cellular oxidative damage induced by hydrogen peroxide (H_2_O_2_). The cells were seeded in 96-well plates at a density of 1 × 10^4^ cells per well and incubated for 24 h to allow for proper attachment and cell adherence. After overnight incubation, different concentrations of various nanoparticles, including MSN, MSN@Uri, RBC@MSN@Uri, SeMSN, SeMSN@Uri, and RBC@SeMSN@Uri, were added to the wells at concentrations ranging from 0 to 160 μg/mL. The cells were then incubated with the nanoparticles for 4 h to ensure adequate interaction between the cells and the nanoparticles. Following this incubation period, H_2_O_2_ was added to each well at a final concentration of 100 μM to induce oxidative stress, and the cells were incubated for an additional 4 h. After exposure to H_2_O_2_, the medium was carefully replaced with fresh culture medium, and the cells were allowed to recover for 24 h. To assess cell viability and determine the extent of oxidative damage, the MTT assay was performed.

### 2.7 Distribution study *in vivo*

To explore the distribution of RBC@SeMSN@Uri, uricase was labeled with Cy7 and intravenously injected into modeling BALB/c mice. Major organs were collected 4 h post-injection and imaged using the IVIS spectral imaging system. Both Uricase and RBC@SeMSN@Uri distributions were examined by injecting Cy7-labeled uricase and collecting major organs 4 h after administration for imaging.

### 2.8 Pharmacokinetics of Uri@RBC *in vivo*

Mice were intravenously injected with Cy7-labeled normal uricase and Cy7-labeled UriSeMSN@RBC containing uricase at the same dose (0.8 mg kg⁻^1^). Blood samples were collected at specified time points to measure and record the fluorescence intensity of Cy7-labeled uricase in the serum.

### 2.9 Construction of acute hyperuricemia models

Healthy male BALB/c mice (20 g) were randomly assigned to four groups: Control, Model, Uricase, and RBC@SeMSN@Uri. Hyperuricemia model were conducted by intraperitoneal injection of 750 mg/kg uric acid and serum uric acid levels were measured to confirm model establishment. Following successful model induction, the Uricase and RBC@SeMSN@Uri groups received intravenous injections of 10 mg/kg of Uricase or RBC@SeMSN@Uri at 0 h. A second dose of 750 mg/kg uric acid was administered intraperitoneally at 11, 23, and 47 h post-initial injection. Blood samples were collected 1 h after each second injection (at 12, 24, and 48 h), and serum uric acid concentrations were analyzed to assess treatment efficacy and monitor hyperuricemia progression.

### 2.10 Construction of diet-induced chronic hyperuricemia

Chronic hyperuricemia was induced in male SD rats (250 g) by administering 10% fructose water for 8 weeks. Blood uric acid levels were measured weekly to confirm hyperuricemia, while blood urea nitrogen (BUN) and creatinine (CREA) levels were also assessed weekly to monitor renal damage. After 8 weeks, fructose feeding was stopped, and uric acid levels were measured again in the ninth week.

Following successful model induction, the Uricase and RBC@SeMSN@Uri groups received intravenous injections of 10 mg/kg Uricase or RBC@SeMSN@Uri at 0 h, coinciding with the cessation of fructose feeding. Blood samples were collected at 6, 12, 24, and 48 h post-treatment. Serum uric acid, BUN, and CREA levels were analyzed to evaluate treatment efficacy and monitor the progression of hyperuricemia.

### 2.11 MSU-induced acute gout model

The gout mice model were conducted following a previously described procedure (Ban et al., 2024). Acute gout models were induced in mice by injecting 20 μL of 40 mg/mL MSU into the right ankle joint under pentobarbital anesthesia. The sham group received 20 μL of saline. Ankle diameters were measured using a Vernier caliper.

### 2.12 Gait analysis

Gait analysis was performed to evaluate limb behavior in mice affected by acute gout induction. Each mouse was placed on a 60 cm × 10 cm track, allowing unrestricted ambulation from one end to the other without external stimulation. Footprints were recorded meticulously, with fore paws dyed in red and hind paws in blue. Three independent observers, blinded to the experimental groups, conducted the acquisition of outcome measures.

### 2.13 Histological analysis

After fixation of mouse knee specimens in 4% paraformaldehyde for 24 h and subsequent decalcification in a 15% ethylenediaminetetraacetate (EDTA)-buffered saline solution for 1 month, the samples were dehydrated, rendered transparent, and embedded in paraffin, resulting in 3 µm thick sections. These sections were stained with hematoxylin and eosin (H&E) to assess inflammatory infiltration within the synovium. Image acquisition was conducted using a light microscope.

## 3 Results and discussion

### 3.1 Synthesis and characterization of RBC@SeMSN@Uri

**FIGURE 2:**
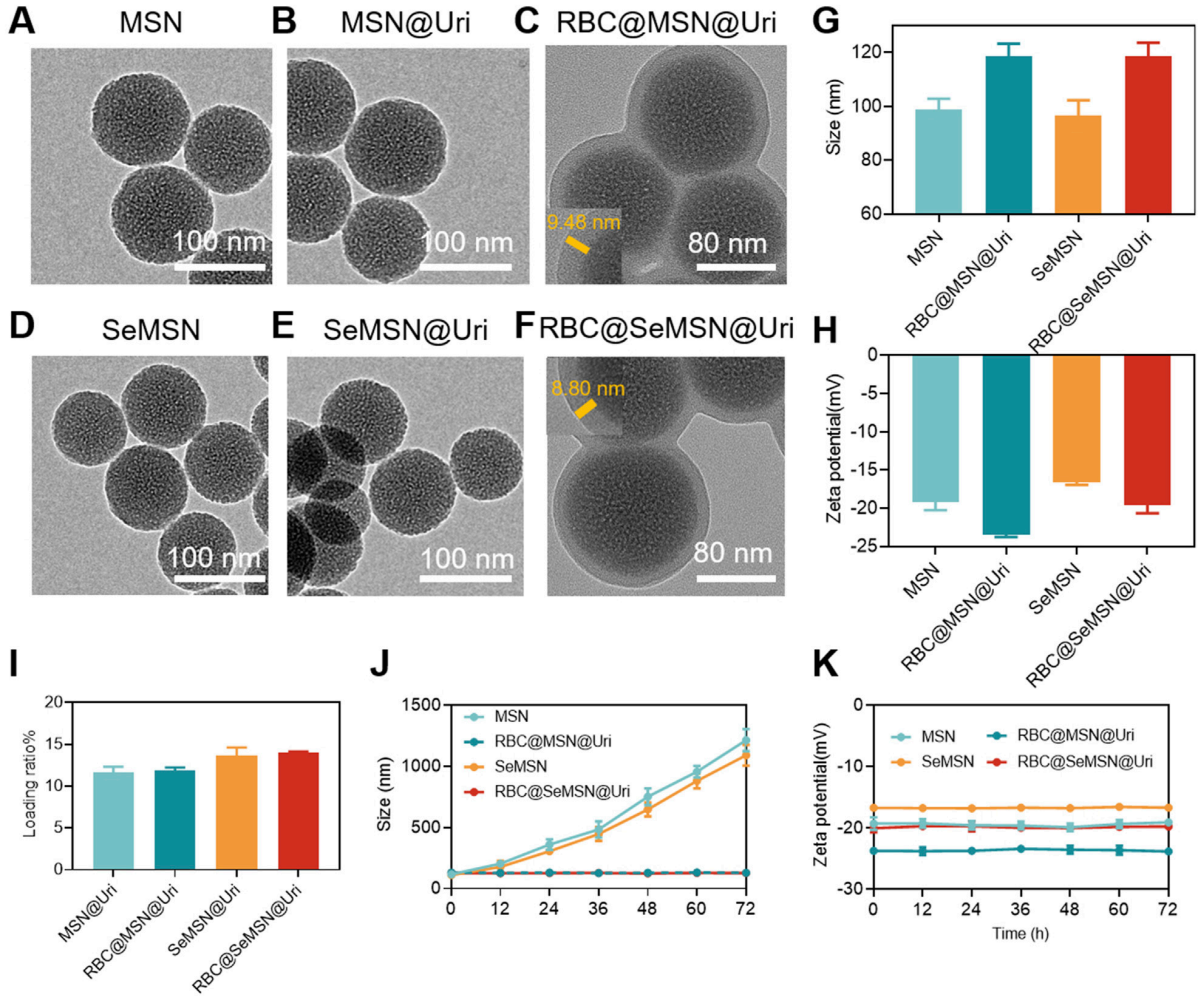
Synthesis and characterization of the RBC@SeMSN@Uri biomimetic self-cascade catalysis bioreactor. **(A)** TEM images of MSN, **(B)** MSN@Uri, **(C)** RBC@MSN@Uri, **(D)** SeMSN, **(E)** SeMSN@Uri, and **(F)** RBC@ SeMSN@Uri, Scale bar = 100 nm. **(G)** Hydrodynamic sizes and **(H)** zeta potentials of MSN, RBC@MSN@Uri, SeMSN and RBC@SeMSN@Uri nanoparticle (n = 3, means ± SEM). **(I)** Uricase loading rate of MSN@Uri, RBC@MSN@Uri, SeMSN@Uri and RBC@SeMSN@Uri nanoparticle (n = 3, means ± SEM). **(J)** Hydrodynamic size and **(K)** zeta potentials of MSN, RBC@MSN@Uri, SeMSN and RBC@SeMSN@Uri after storage at room temperature in 10% fetal bovine serum-containing medium for 72 h, respectively. The data was obtained from DLS. Data are presented as means ± SEM (n = 3).

Mesoporous Silica Nanoparticles (MSN) and Diselenide-bridged MSN (SeMSN) were synthesized using a modified sol–gel method as described in previous studies (Shao et al., 2018; Shao et al., 2020; Zhang et al., 2021; Zhang et al., 2023), achieving a high selenium density of 9.9% in SeMSNs. Transmission Electron Microscopy (TEM) images of MSN and SeMSN showed monodisperse, spherical particles with average sizes of 70 nm and 68 nm, respectively, both featuring numerous small pores (Figures 2A, D). N_2_ adsorption-desorption isotherms indicated that the Brunauer-Emmett-Teller (BET) surface area, total pore volume, and average pore size of SeMSN were 577.069 m^2^/g, 1.206 cm³/g, and 8.361 nm, respectively (Supplementary Figure S1). Following synthesis, uricase was loaded onto MSN and SeMSN, achieving high loading rates of 11.3 wt% and 13.6 wt%, respectively (Figure 2I).

To enhance circulation time and reduce immunogenicity, the uricase-loaded MSN/SeMSN (MSN@Uri/SeMSN@Uri) were coated with red blood cell (RBC) membranes, as previously reported (Yang et al., 2024) (Figure 1). Dynamic light scattering analysis revealed that both RBC@MSN@Uri and RBC@SeMSN@Uri displayed hydrodynamic sizes approximately 20–22 nm larger than the uncoated MSN or SeMSN nanoparticles (96.4 nm and 98.8 nm, respectively), confirming the addition of an RBC bilayer membrane to the polymeric cores (Figure 2G). TEM images further demonstrated a distinctive core-shell structure for RBC@MSN@Uri and RBC@SeMSN@Uri, distinguishing them from the bare MSN/SeMSN cores and visually confirming the membrane coating (Figures 2B, C, E, F). Zeta potential analysis showed that RBC-coated nanoparticles exhibited a 4 mV lower zeta potential compared to uncoated MSN or SeMSN, further evidencing the successful RBC membrane coating (Figure 2H). To assess colloidal stability, MSN, SeMSN, RBC@MSN@Uri, and RBC@SeMSN@Uri were dispersed in a 10% fetal bovine serum-containing medium for 72 h. Uncoated nanoparticles quickly aggregated to the microscale, while the RBC-coated nanoparticles maintained stable particle size and zeta potential (Figures 2J, K), demonstrating excellent colloidal stability and monodispersity in a complex hematological environment.

### 3.2 RBC@SeMSN@Uri is a self-cascade bioreactor for uric acid and H_2_O_2_ degradation *in vitro*

**FIGURE 3:**
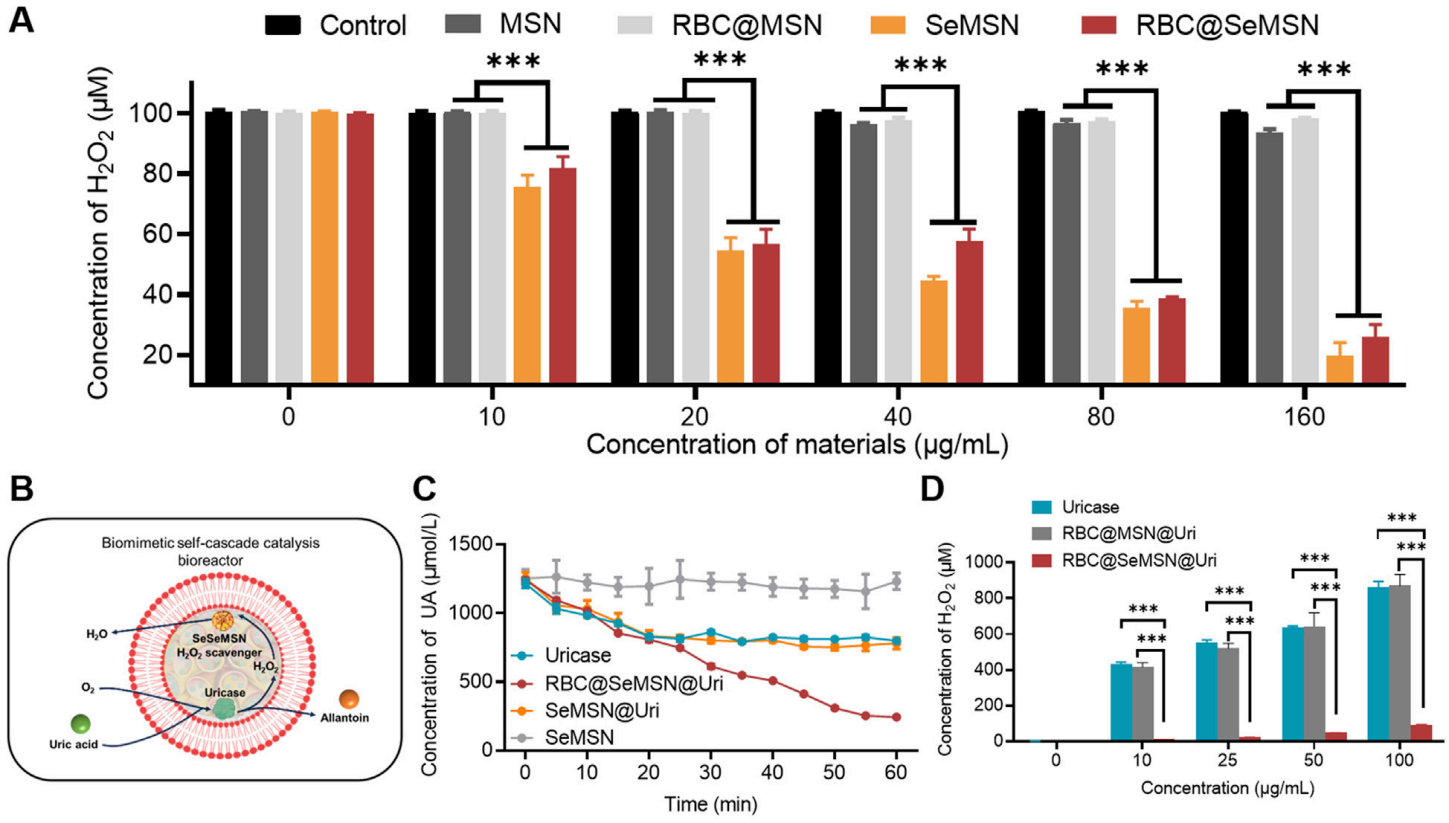
H_2_O_2_ degradation activity of SeMSN and RBC@SeMSN, as well as the self-cascade degradation H_2_O_2_ of RBC@SeMSN@Uri. **(A)** H_2_O_2_ degradation activity of MSN, RBC@MSN, SeMSN, and RBC@ SeMSN. **(B)** Schematic diagram of the self-cascade degradation H_2_O_2_ of RBC@SeMSN@Uri. The H_2_O_2_ generated *in situ* by uricase is immediately utilized by SeMSN for degradation H_2_O_2_ within the erythrocyte membrane, highlighting the cooperative interaction between SeMSN and uricase in the self-cascade of RBC@SeMSN@Uri. **(C)** Concentration of uric acid in serum when treated with 0.01 mg/mL uricase or RBC@SeMSN@Uri *in vitro*, highlighting the role of the RBC membrane in enhancing uricase stability. **(D)** Concentration of accumulated H_2_O_2_ was detected after uric acid degradation by different concentration of uricase or RBC@SeMSN@Uri *in vitro* (n = 3). Data are means ± SEM (***p < 0.001 by one-way analysis of variance (ANOVA) with Tukey’s multiple comparison test).

To investigate the efficiency of hydrogen peroxide (H_2_O_2_) degradation, we examined MSN, SeMSN, RBC@MSN and RBC@SeMSN nanoparticles. After co-incubation with 100 μM H_2_O_2_ at varying concentrations, we found that the degradation of H_2_O_2_ increased with higher concentrations of SeMSN and RBC@SeMSN (Figure 3A), demonstrating their high efficiency in breaking down H_2_O_2_. In contrast, MSN and RBC@MSN did not show any ability to degrade H_2_O_2_. Notably, the RBC coating on SeMSN did not significantly affect its H_2_O_2_ scavenging activity, as both SeMSN and RBC@SeMSN exhibited similar degradation efficiencies (Figure 3A). These results confirm that SeMSN and RBC@SeMSN effectively degrade H_2_O_2_, unlike MSN and RBC@MSN, which lack this capability. This underscores the distinct degrading H_2_O_2_ properties of selenium-based nanoparticles. Next, we investigated whether loading uricase into RBC@SeMSN could enable a self-cascade degradation of H_2_O_2_ produced during the catalytic degradation of uric acid by uricase (Figure 3B). The RBC@SeMSN@Uri complex demonstrated superior uric acid degradation in serum compared to free uricase, completely degrading uric acid within 60 min, while free uricase only achieved a 30% degradation in the same timeframe (Figure 3C). This suggests that the RBC coating helps preserve the enzymatic activity and efficiency of uricase in degrading uric acid. Furthermore, RBC@SeMSN@Uri effectively degraded H_2_O_2_ generated *in situ* by uricase, with SeMSN immediately utilizing the H_2_O_2_ for its GPx-like activity to convert it into H_2_O (Figure 3D). This demonstrates a cooperative interaction between SeMSN and uricase, enhancing the self-cascade degradation process. Overall, RBC@SeMSN@Uri serves as a self-cascade bioreactor, maintaining high stability, preserving enzymatic activity, and achieving efficient degradation of both uric acid and H_2_O_2_.

### 3.3 RBC@SeMSN@Uri exhibits excellent biocompatibility, anti-inflammatory effects, and protection against oxidative stress

**FIGURE 4:**
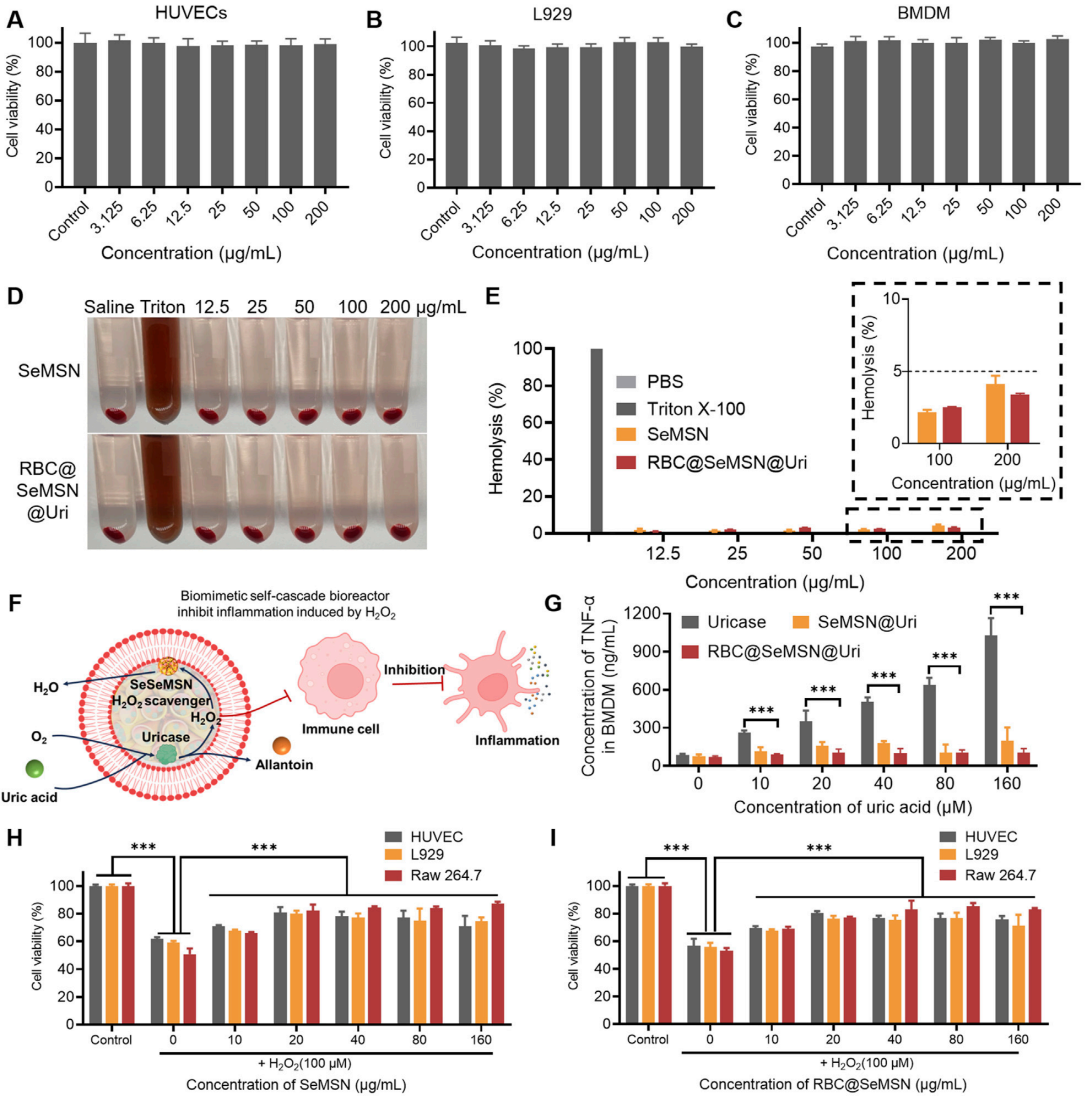
*In vitro* cytotoxicity, antioxidant, and anti-inflammatory effects of RBC@SeMSN@Uri. **(A)** Cell viability of HUVECs, **(B)** L929 cells, and **(C)** BMDM cells treated with RBC@SeMSN@Uri. **(D)** Hemolysis images of 2% RBCs after 24 h of treatment with RBC@SeMSN@Uri. **(E)** Hemolysis analysis of 2% RBCs induced by RBC@SeMSN@Uri compared with Triton X-100. **(F)** Schematic illustration showing how RBC@SeMSN@Uri reduces H_2_O_2_-induced inflammation; H_2_O_2_ generated *in situ* by uricase is immediately scavenged by SeMSN, reducing H_2_O_2_-induced inflammation. **(G)** Concentration of TNF-α in BMDM cells induced by H_2_O_2_ produced by uricase catalyzing the degradation of uric acid. **(H)** RBC@SeMSN@Uri and **(I)** SeMSN reduce H_2_O_2_-induced cell damage. n = 3, Data are presented as mean ± SEM (***p < 0.001 by one-way ANOVA with Tukey’s multiple comparison test).

Before using the RBC@SeMSN@Uri nano-bioreactor for hyperuricemia and gout treatment, its cellular behaviors were first examined *in vitro*, including cytotoxicity, H_2_O_2_ degradation, and anti-inflammatory effects. As shown in Figures 4A–C and the Supplementary Figure S2, RBC@SeMSN@Uri demonstrated good biocompatibility with various cell lines, including human umbilical vein endothelial cells (HUVECs), L-929 cells, Raw 264.7 cells, and bone marrow-derived macrophages (BMDMs), across a concentration range of 0–200 µg/mL. The hemolytic activity of RBC@SeMSN@Uri was further assessed by co-incubating the bioreactor with 2% red blood cells (RBCs) for 24 h. Unlike the positive control group treated with Triton X-100, which showed significant hemolysis, the SeMSN and RBC@SeMSN@Uri groups exhibited minimal hemolysis, with a ratio of less than 5% even at 200 µg/mL (Figures 4D, E). These findings suggest that the RBC@SeMSN@Uri bioreactor possesses good *in vitro* compatibility.

To simulate a hyperuricemia therapy environment, BMDM cells were exposed to different concentrations of uric acid (UA) to study inflammatory stress (Figure 4F). Co-incubation with uricase alone increased levels of inflammatory markers TNF-α and IL-6 in BMDMs as UA concentrations rose (Figure 4G; Supplementary Figure S3). In contrast, the RBC@SeMSN@Uri nanoreactor significantly reduced these inflammatory factors by rapidly degrading H_2_O_2_ generated *in situ* by uricase (Figure 4G). Furthermore, the ability of RBC@SeMSN@Uri to suppress ROS-induced apoptosis was evaluated using an MTT assay in HUVECs, L-929, and Raw 264.7 cells. After pretreatment with SeMSN and RBC@SeMSN@Uri nanoparticles, cell viability significantly increased when cells were exposed to exogenous H_2_O_2_, compared to untreated HUVECs, L-929, and Raw 264.7 cells (Figures 4H, I; Supplementary Figure S4). This result demonstrates that SeMSN and RBC@SeMSN@Uri has a significant protective effect on cells and inhibits apoptosis under oxidative stress. Overall, these results suggest that RBC@SeMSN@Uri possesses excellent biocompatibility, effectively degrades H_2_O_2_, reduces inflammation, and protects against oxidative stress, making it a promising candidate for therapeutic applications in hyperuricemia and gout.

### 3.4 Therapeutic effects of RBC@SeMSN@Uri in acute and chronic hyperuricemia models

**FIGURE 5:**
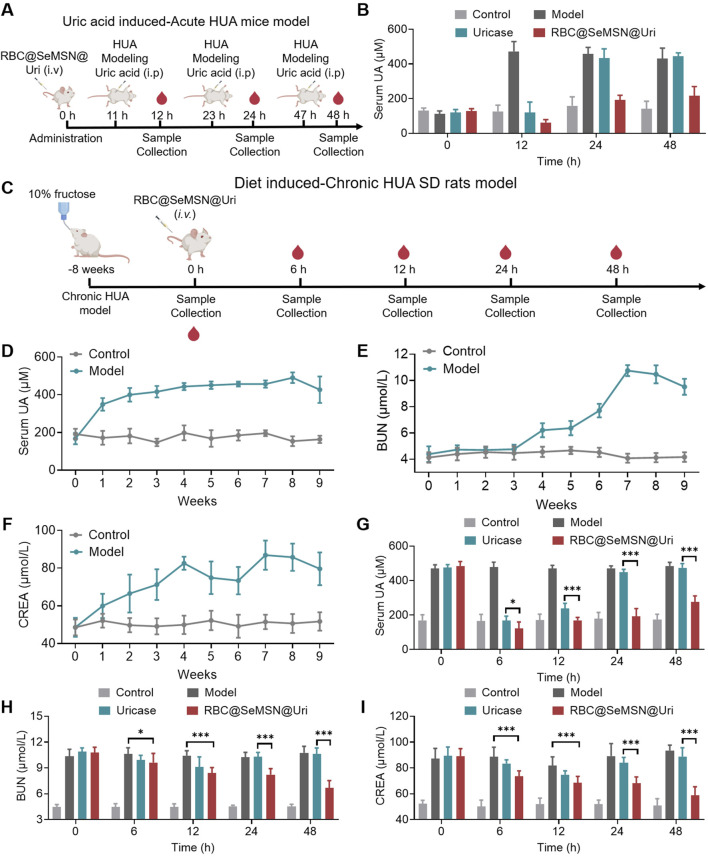
Therapeutic effects of RBC@SeMSN@Uri in acute and chronic hyperuricemia models. **(A)** Schematic illustrating the construction of an acute hyperuricemia (HUA) mouse model induced by uric acid and demonstrating the prolonged circulation of RBC@SeMSN@Uri following a single intravenous injection for treatment. **(B)** Serum uric acid (UA) levels in acute HUA mice measured at 12, 24, and 48 h post-treatment with RBC@SeMSN@Uri (n = 4). **(C)** Schematic depicting the creation of a chronic hyperuricemia (HUA) rat model via a 10% fructose diet for 2 months, followed by treatment with RBC@SeMSN@Uri after a single intravenous injection. **(D)** Serum UA levels in chronic HUA rats monitored over 8 weeks on a 10% fructose diet (n = 4). **(E)** Levels of blood urea nitrogen (BUN) and **(F)** creatinine (CREA) in chronic HUA rats measured at different time points (1, 2, 3, 4, 5, 6, 7, and 8 weeks) (n = 4).**(G)** Concentration of UA in serum when treated with uricase or RBC@SeMSN@Uri in chronic HUA rats at different time points (0, 6, 12, 24, and 48 h) (n = 4). **(H)** Levels of BUN and **(I)** CREA in chronic HUA rats treated with either uricase or RBC@SeMSN@Uri, measured at 0, 6, 12, 24, and 48 h (n = 4). Data are presented as mean ± SEM (***p < 0.001 by one-way ANOVA with Tukey’s multiple comparison test).

Given the excellent biocompatibility, uric acid degradation, and oxidative stress protection of RBC@SeMSN@Uri *in vitro*, we hypothesized that RBC@SeMSN@Uri could provide a long-lasting uric acid-lowering effect for hyperuricemia treatment. To test this, we first developed an acute hyperuricemia mouse model by injecting uric acid intraperitoneally to rapidly elevate blood UA levels. RBC@SeMSN@Uri was administered at 0 h, followed by additional uric acid injections at 11, 23, and 47 h to evaluate its prolonged circulation and therapeutic effects. UA levels were measured 1 h after each injection (Figure 5A). A single dose of RBC@SeMSN@Uri maintained a UA-lowering effect for up to 48 h, while free uricase lost its effectiveness within 24 h (Figure 5B). This demonstrates the sustained uric acid-lowering capability of RBC@SeMSN@Uri, highlighting the role of the RBC coating in preserving enzymatic activity.

To further assess the sustained effect of RBC@SeMSN@Uri, we conducted a chronic hyperuricemia model in SD rats fed a 10% fructose diet for 8 weeks (Figure 5C). After 4 weeks, blood UA levels significantly increased to over 440 μM and remained elevated throughout the study. Even after discontinuing the fructose diet, high UA levels persisted for over a week (Figure 5D). Given the potential adverse effects of hyperuricemia on kidney function, we measured serum biochemical markers such as blood urea nitrogen (BUN) and creatinine (CREA). BUN levels increased from weeks 4–7, peaking at 10.75 μM, and remained high through week 8, persisting for more than a week after stopping the diet (Figure 5E). Similarly, CREA levels rose from weeks 1–4 and stayed elevated from weeks 4–8, even after the diet ended (Figure 5F). These elevated BUN and CREA levels confirm the successful establishment of the chronic hyperuricemia model, indicating not only increased UA levels but also kidney damage, closely mimicking clinical symptoms of hyperuricemia. In the chronic hyperuricemia model, a single intravenous injection of RBC@SeMSN@Uri rapidly reduced blood UA levels and maintained this reduction for up to 48 h, whereas free uricase lost its effect within 24 h (Figure 5G). After 48 h of RBC@SeMSN@Uri treatment, BUN levels significantly dropped to 6.68 μM, compared to 10.61 μM in the free uricase group, which was similar to the hyperuricemia model group (Figure 5H). CREA levels also decreased to 58.81 μM, approaching those of the control group (50.93 μM), while the free uricase group had a CREA level of 88.79 μM, close to that of the hyperuricemia model group (93.44 μM) (Figure 5I). These findings confirm that RBC@SeMSN@Uri effectively reduces uric acid levels and mitigates kidney damage in hyperuricemia models, achieving a prolonged and efficient therapeutic effect in hyperuricemia treatment.

### 3.5 Therapeutic effect of RBC@SeMSN@Uri on MSU-Induced acute gouty arthritis

**FIGURE 6:**
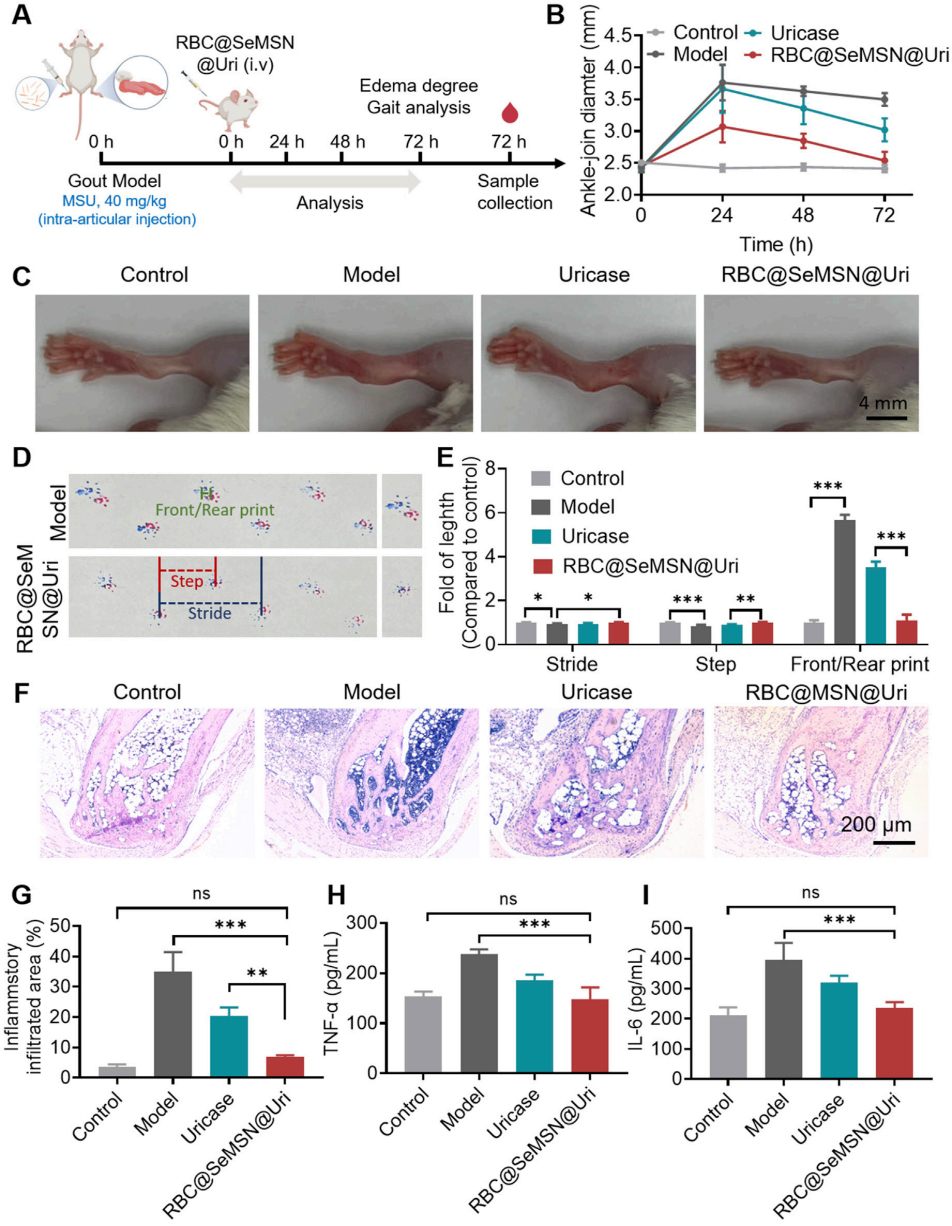
Therapeutic Effect of RBC@SeMSN@Uri on MSU-Induced Acute Gouty Arthritis. **(A)** Schematic representation of the acute gouty arthritis mouse model induced by monosodium urate, followed by treatment with RBC@SeMSN@Uri via a single intravenous injection. **(B)** Swelling degree of the ankle joints at 0, 24, 48, and 72 h post-modeling in Control, Model, Uricase, and RBC@SeMSN@Uri groups (n = 4). **(C)** Representative images of ankle joints at 72 h post-modeling (n = 4). **(D)** Walking imprint analysis at 72 h post-modeling, showing stride length (blue), step length (red), and front/rear paw print lengths (green). Fore paw prints are red, and hind paw prints are blue (n = 4). **(E)** Quantification of stride length, step length, and front/rear paw print lengths (n = 4). **(F)** H&E staining images of ankle joint areas in Control, Model, Uricase, and RBC@SeMSN@Uri groups at 72 h post-modeling (n = 4). **(G)** Quantification of inflammatory infiltrated area percentage at 72 h post-modeling (n = 4). **(H)** Inflammatory factor levels of TNF-α and **(I)** IL-6 in the synovial fluid of mouse ankle joints (n = 4). Data are expressed as mean ± SEM. Statistical significance: *p < 0.05, **p < 0.01, ***p < 0.001 by one-way ANOVA with Tukey’s multiple comparison test.

Encouraged by the strong uric acid-lowering effects of RBC@SeMSN@Uri in a hyperuricemia model, we investigated its therapeutic potential in gouty arthritis. We established an acute gouty arthritis mouse model by injecting monosodium urate (MSU) crystals into the joint cavity (Figure 6A). At 24 h post-injection, the ankle joints of model mice swelled to 3.76 mm, with swelling persisting at 3.50 mm at 72 h. Treatment with RBC@SeMSN@Uri reduced joint swelling to 2.53 mm at 72 h, comparable to the control group at 2.42 mm (Figures 6B, C). In contrast, mice treated with free uricase showed persistent swelling of 3.02 mm (Figures 6B, C). These results demonstrate that RBC@SeMSN@Uri significantly alleviated joint swelling in the acute gouty arthritis model, showing superior therapeutic efficacy compared to free uricase.

Next, gait analysis was performed to evaluate locomotor ability and pain severity in mice after RBC@SeMSN@Uri treatment. As expected, mice with gouty arthritis had significantly shorter steps than the normal control group, reflecting impaired joint mobility and increased discomfort due to inflammation and pain (Figures 6D, E). Following treatment with RBC@SeMSN@Uri, step length improved, indicating reduced joint pain and partial recovery of mobility. In normal mice, front and rear paw prints typically overlapped, showing coordinated movement, whereas gouty mice exhibited a clear separation between these prints, demonstrating altered gait patterns. Remarkably, RBC@SeMSN@Uri treatment reduced this separation (Figures 6D, E), suggesting a restoration of normal gait. In contrast, mice treated with free uricase continued to show significant separation between paw prints (Figures 6D, E). These findings indicate that RBC@SeMSN@Uri offers a superior therapeutic effect over free uricase, enhancing recovery and reducing pain in mice with MSU-induced acute gout.

Next, H&E staining was performed to evaluate the inflammation and structural changes in the ankle joints after RBC@SeMSN@Uri treatment. As shown in Figure 6F, the model group exhibited significant inflammatory cell infiltration, cartilage erosion, and bone damage, which are characteristic symptoms of gouty arthritis. In contrast, mice treated with RBC@SeMSN@Uri showed a marked reduction in inflammatory cells, nearly reaching levels seen in the control group. The articular cartilage in the treated group showed substantial recovery, with improved joint structure, intact bone, and minimal inflammatory infiltration, closely resembling normal cartilage. Meanwhile, the uricase-treated group still exhibited notable inflammatory infiltration, with an infiltrated area rate of 20.03%, although there was some improvement in joint condition (Figures 6F, G). These results highlight the superior anti-inflammatory effects of RBC@SeMSN@Uri compared to free uricase. Consistent with these findings, the levels of inflammatory factors TNF-α and IL-6 in the synovial fluid of the RBC@SeMSN@Uri-treated mice significantly decreased from 237.6 pg/mL and 396.0 pg/mL in the model group to 147.4 pg/mL and 235.0 pg/mL, respectively, nearly returning to normal levels (Figures 6H, I). Overall, these results demonstrate that RBC@SeMSN@Uri effectively reduces joint swelling and inflammation in MSU-induced gout, highlighting its therapeutic potential.

### 3.6 Biodistribution and pharmacokinetic of RBC@SeMSN@Uri

**FIGURE 7:**
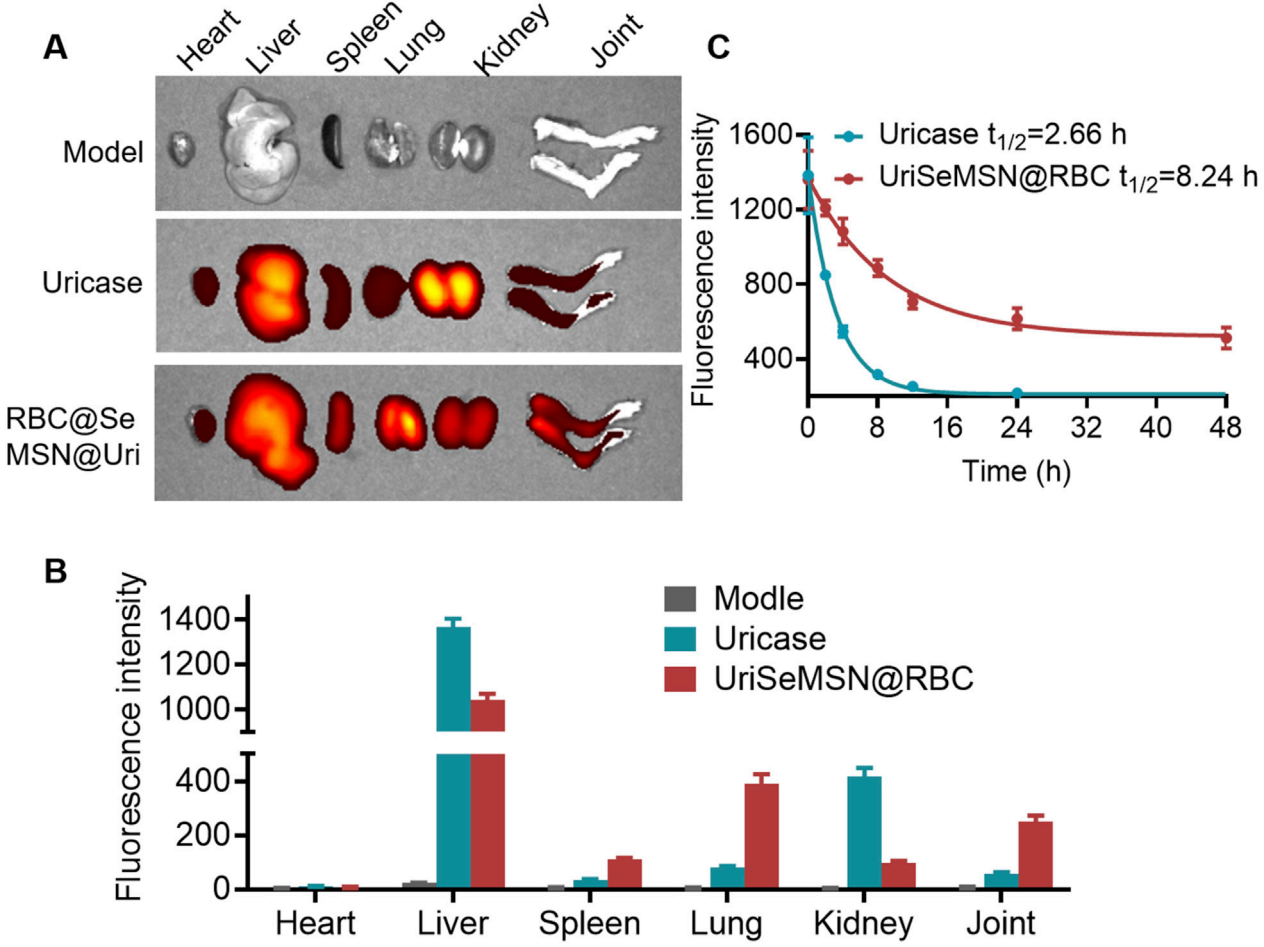
Biodistribution and pharmacokinetic of RBC@SeMSN@Uri on MSU-Induced Acute Gouty Arthritis mice. **(A)** Biodistribution of RBC@SeMSN@Uri (Cy7-labeled uricase) in major organs of MSU-induced acute gouty arthritis mice 6 h post-injection. **(B)** Quantitative analysis of Cy7-labeled RBC@SeMSN@Uri in major organs. **(C)** Pharmacokinetic profile of Cy7-labeled RBC@SeMSN@Uri following intravenous injection in the gouty arthritis model (n = 4).

Before RBC@SeMSN@Uri can be widely used clinically, it is essential to assess its biodistribution and potential side effects in mice. We conducted intravenous injections of Cy7-labeled RBC@SeMSN@Uri and Cy7-labeled free uricase to monitor fluorescence in various organs, including the lungs, heart, spleen, kidneys, and liver. As shown in Figure 7A, RBC@SeMSN@Uri nanoparticles primarily accumulated in the liver within 6 h, likely due to the liver’s role in filtering nanoparticles. Notably, RBC@SeMSN@Uri also accumulated in the joints at levels 4.47 times higher than free uricase, indicating enhanced targeting of inflamed areas, which is beneficial for treating gouty arthritis (Figures 7A, B). Pharmacokinetic (PK) profiles, summarized in Figure 7C, revealed that the elimination half-life (t_1/2_) of free uricase was approximately 2.66 h, while RBC@SeMSN@Uri had a significantly extended half-life of 8.24 h, about 3.1 times longer. This suggests RBC@SeMSN@Uri remains in circulation much longer than free uricase. Free uricase was nearly undetectable 16 h after administration, whereas RBC@SeMSN@Uri levels remained high even 48 h post-administration, demonstrating a prolonged circulation time. The extended half-life and targeted biodistribution of RBC@SeMSN@Uri enable it to maintain therapeutic levels for a longer period, reducing the need for frequent dosing in chronic conditions like hyperuricemia and gout.

### 3.7 Biosafety of RBC@SeMSN@Uri *in vivo*

**FIGURE 8:**
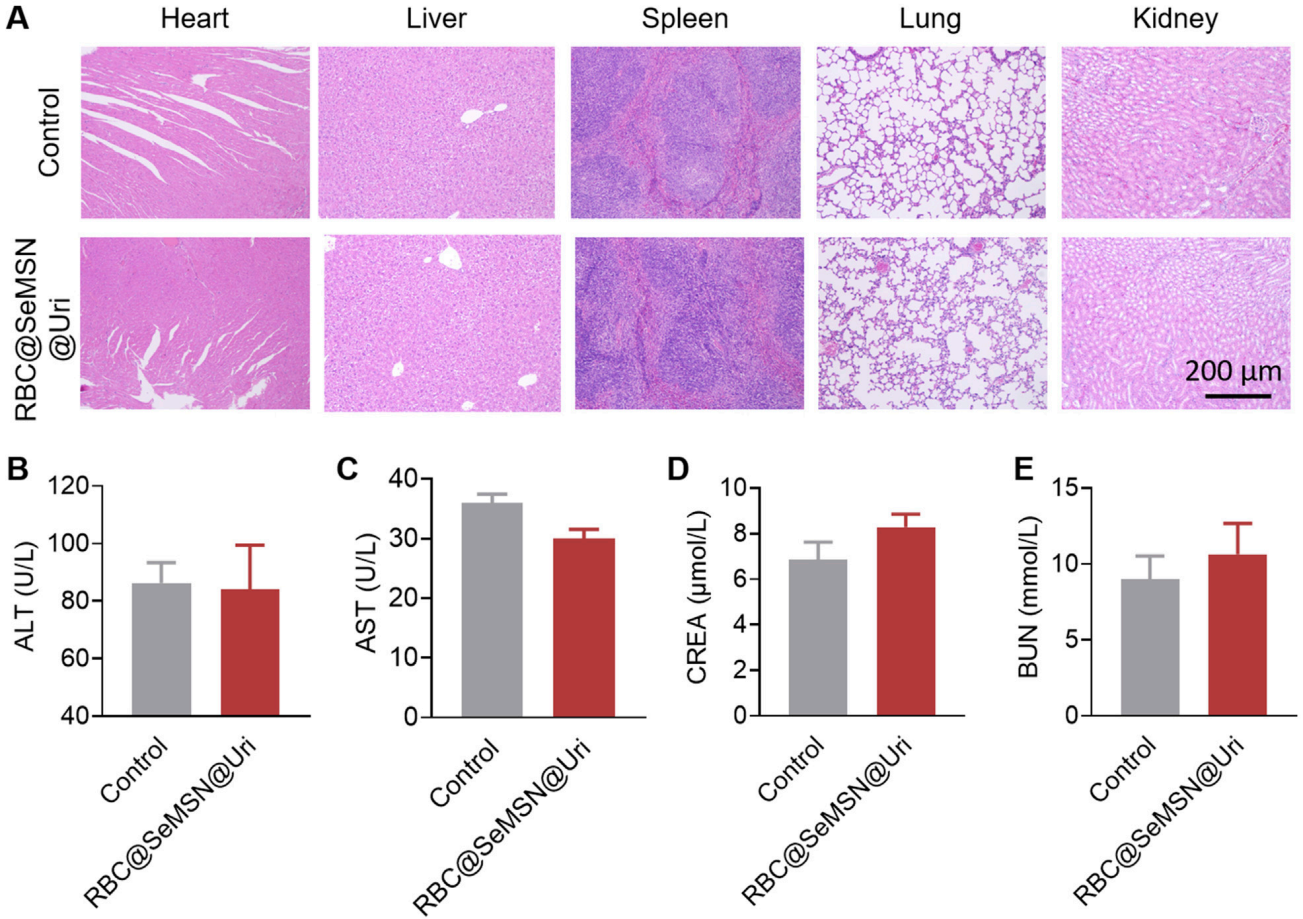
Pathological analysis of major organs and blood biochemical indices in normal mice following RBC@SeMSN@Uri injection. **(A)** H&E staining of heart, liver, spleen, lung, and kidney tissues from normal mice 7 days post-injection. Scale bars, 200 μm. **(B–E)** Blood levels of **(B)** alanine transaminase (ALT), **(C)** aspartate aminotransferase (AST), **(D)** blood urea nitrogen (BUN), and **(E)** creatinine (CREA) in normal mice after 7 days post-injection. (n = 4).

Evaluating the biosafety of RBC@SeMSN@Uri is essential to ensure it does not cause side effects or organ toxicity. Throughout the study, no deaths or signs of acute toxicity were observed in mice treated with RBC@SeMSN@Uri, indicating its safety. To further assess this, serum biochemistry and histological evaluations using H&E staining were performed. Histological analysis revealed no pathological changes or tissue damage in major organs, including the heart, liver, spleen, lung, and kidney (Figure 8A). The serum biochemistry analysis also showed no significant differences in liver or kidney function between the RBC@SeMSN@Uri-treated group and the control group 7 days post-injection. Liver markers, such as alanine transaminase (ALT) and aspartate aminotransferase (AST), as well as kidney markers, including creatinine (CRE) and blood urea nitrogen (BUN), remained within normal ranges, suggesting no hepatic or renal toxicity (Figures 8B–E). These findings indicate that RBC@SeMSN@Uri has a favorable biosafety profile with minimal systemic toxicity, making it a promising therapeutic candidate for managing hyperuricemia and gout without harming major organs.

## 4 Conclusion

In summary, a novel RBC-coated biomimetic self-cascade bioreactor (RBC@SeMSN@Uri) was designed and synthesized for the treatment of hyperuricemia and gout. The bioreactor integrates uricase and selenium-based nano-scavenger (SeMSN) within red blood cell membranes to convert uric acid to allantoin and H_2_O_2_, while SeMSN further transform H_2_O_2_ into water, mitigating oxidative damage and inflammation. *In vitro* assays demonstrated excellent biocompatibility, anti-inflammatory effects, and protection against oxidative stress. *In vivo* models showed that RBC@SeMSN@Uri effectively reduces uric acid levels and alleviates symptoms of hyperuricemia and gout, with prolonged circulation and targeted biodistribution. The bioreactor significantly outperforms free uricase in terms of therapeutic duration and efficacy. Our approach, leveraging RBC-coated uricase and selenium-based nano-scavenger, addresses key limitations of traditional therapies, offering an effective, safer, and enhanced treatment for gout and related conditions.

## Data Availability

The original contributions presented in the study are included in the article/Supplementary Material, further inquiries can be directed to the corresponding authors.
