# Supplementary material for: Biomimetic bioreactor for potentiated uricase replacement therapy in hyperuricemia and gout
Source: Front Bioeng Biotechnol. 2025 Jan 7;12:1520663. doi: 10.3389/fbioe.2024.1520663 (PMC11746906; doi:10.3389/fbioe.2024.1520663)
Supplement: Supplementary file 1 [file DataSheet1.pdf]

Figure Captions

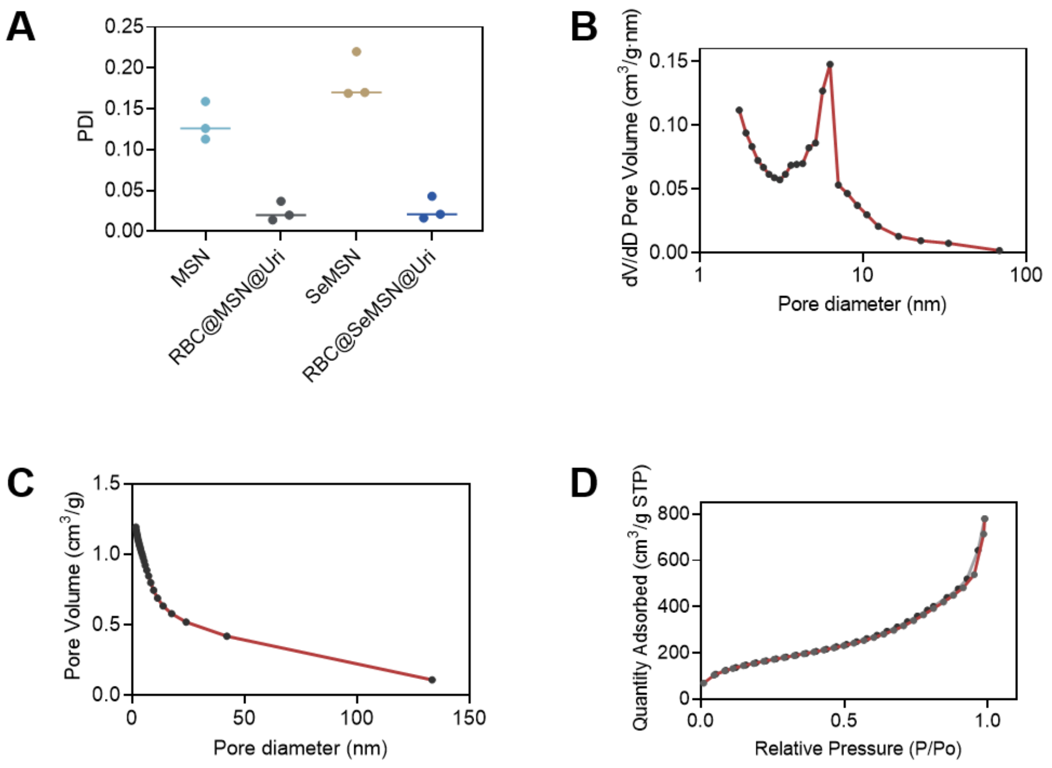

Figure S1. characterization of the SeMSN. (A)PDI of MSN, RBC@MSN@Uri, SeMSN and RBC@SeMSN@Uri nanoparticle (n=3, means±SEM). (B) N2 sorption isotherm, (C) pore size distribution and (D)surface area of SeMSN.

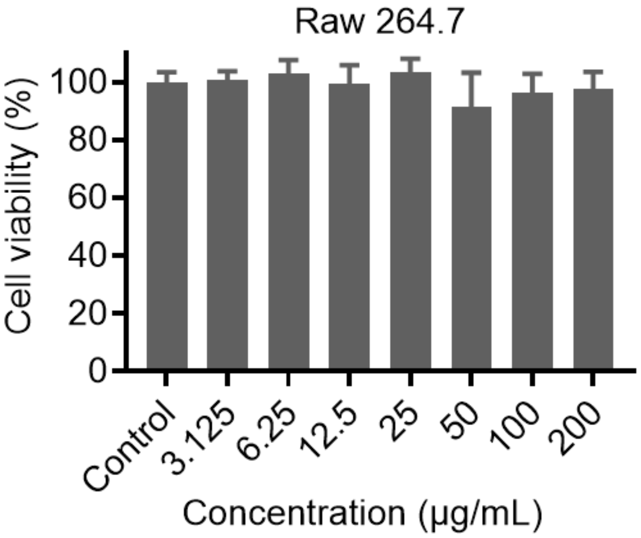

Figure S2. Cell viability of Raw 264.7 cells treated with RBC@SeMSN@Uri.

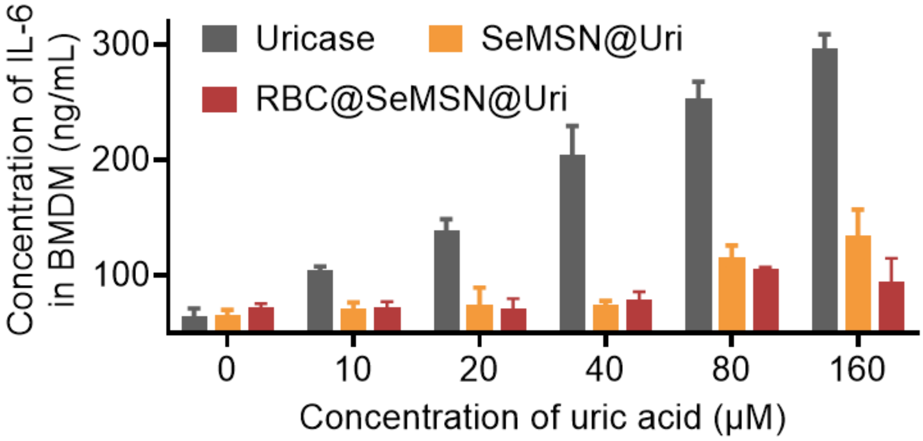

Figure S3. Concentration of IL-6 in BMDM cells induced by H_2O_2 produced by uricase catalyzing the degradation of uric acid.

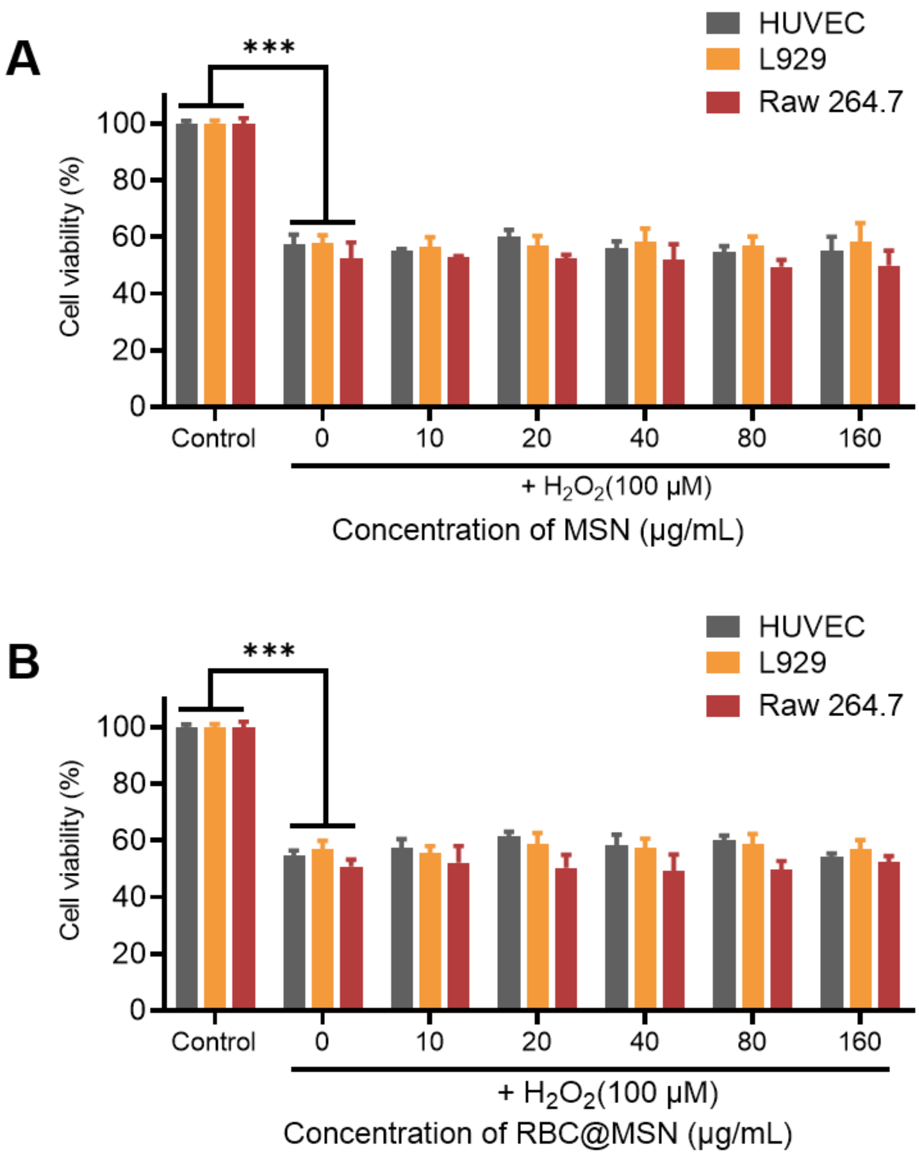

Figure S4. (**A**) MSN and (**B**) RBC@MSN@Uri reduce H_2O_2-induced cell damage. n = 3, Data are presented as mean ± SEM (***$p < 0.001$ by one-way ANOVA with Tukey's multiple comparison test).

1.1 Materials

Uricase was purchased from Nanjing Jiancheng Co., Ltd. (Nanjing, China). Uric acid (UA) was purchased from Adamas Co., Ltd. (Shanghai, China). Fetal bovine serum (FBS), penicillin and streptomycin, BCA Protein Assay Kits were purchased from Invitrogen (USA). TNF-α and IL-6 ELISA kits were purchased from Biolegend (USA). H_2O_2 test kit was purchased from Beyotime Co., Ltd. (Shanghai, China).

1.2 Preparation of RBC-membrane-derived vesicles.

RBC-membrane-derived vesicles were prepared following the method outlined in previous literature(Hu et al., 2011). Whole blood was collected from male BALB/c mice(5–8 weeks old, procured from Hunan SJA Laboratory Animal Co. Ltd). The blood was first centrifuged at 500g for 5 minutes at 4°C to separate the plasma and buffy coat. The remaining packed red blood cells (RBCs) were washed three times with ice-cold 1× PBS to remove residual plasma components. Next, the RBCs were subjected to hemolysis in a hypotonic solution to lyse the cells. Specifically, the washed RBCs were suspended in 0.25× PBS and incubated in an ice bath for 20 minutes. This suspension was then centrifuged at 500g for another 5 minutes to separate the cell membranes from the cytoplasmic contents. The supernatant, which contained released hemoglobin, was carefully discarded, and the light pink pellet of RBC membranes was collected. These membranes were washed twice with 1× PBS to ensure purity. To produce RBC-membrane-derived vesicles, the cleaned RBC ghosts were sonicated in a capped glass vial for 5 minutes at a frequency of 42 kHz and a power of 100 W. This sonication process led to the formation of vesicles by breaking the RBC membranes into small, closed structures, preserving the native membrane proteins and lipids. These RBC-membrane-derived vesicles were then ready for further experimentation and store at-80°C.

1.3 Preparation of SeMSN

The disulfide-bridged mesoporous silica nanoparticles (MSN) were synthesized following a previously described procedure(Shao et al., 2018). Initially, 4.0 g of cetyltrimethylammonium tosylate (CTAT), 1.6 g of triethanolamine ($TEAH_3$), and 200 mL of deionized water were mixed and stirred at 80°C for 1 hour. Following this, a solution of 3.2 g of tetraethyl orthosilicate (TEOS) and 2.4 g of bis[3-(triethoxysilyl)propyl] tetrasulfide (BTESPT) was slowly added dropwise to the surfactant mixture while maintaining the stirring at 80°C for an additional 4 hours at 1000 rpm. The resulting mixture was then subjected to centrifugation to collect the product, which was washed three times with ethanol. To remove the surfactant, the product was refluxed in an ethanol solution containing 1% (w/v) ammonium nitrate (NH_4NO_3) for 12 hours. The disulfide-bridged MSNs were then collected, washed thoroughly, and dried under vacuum. In the next step, 1.0 g of the MSN was dispersed in 250 mL of toluene and subjected to sonication for 1 hour to ensure uniform dispersion. Subsequently, 1.5 mL of 3-chloropropyltrimethoxysilane (CPTMO) was added, and the mixture was refluxed at 80 °C for 24 hours to functionalize the MSN with chloropropyl groups. Finally, the chlorinated MSN was separated by centrifugation, washed multiple times with toluene to remove any unreacted reagents, and dried under vacuum. These chlorinated MSNs were then ready for use in subsequent experiments.

1.4 Cell Viability Assay

L-929 cells, RAW 264.7 cells, HUVECs (1 × 10^4 cells per well) were seeded in 96-well plates and incubated for 24 hours to allow attachment. The medium was then replaced with culture medium containing RBC@SeMSN@Uri at concentrations ranging from 0 to 100 × 10^5/mL. After 12 hours of treatment, the cell monolayers were fixed with 10% (wt/vol) trichloroacetic acid and stained for 1 hour. Excess dye was removed by washing five times with 1% (vol/vol) acetic acid and once with deionized water (ddH2O). The protein-bound dye was dissolved in 10 mM Tris base solution, and the optical density (OD) was measured at 510 nm using a microplate reader.

1.5 Measurement of UA Degradation *in vitro*

The degradation of uric acid (UA) was tracked by recording absorbance changes at 292 nm every 10 minutes with a UV-Visible spectrophotometer. The reaction mixture included Uri or RBC@SeMSN@Uri (20 μg/mL) and UA (100 μM) in PBS buffer (10 mM, pH 7.3) or serum at 37°C.

1.6 Detection of Accumulated H_2O_2 after UA Degradation.

The accumulated H2O2 after UA degradation was detected using a Hydrogen Peroxide Assay Kit. A 1.7 μM UA solution was incubated with varying concentrations of uricase or RBC@SeMSN@Uri (0, 10, 25, 50, 100 μg/mL uricase) for 30 minutes. Following UA degradation catalyzed by natural uricase and RBC@SeMSN@Uri, the RBCs were removed by centrifugation at 500 rpm for 5 minutes. Then, 50 μL of supernatant and 100 μL of test solution were incubated at room temperature for 20 minutes, and the absorbance at 560 nm was measured using a microplate reader. The H2O2 concentration was calculated based on a standard curve.

1.7 Preparation of MSU

Monosodium urate (MSU) was prepared as follows: Initially, 1 g of UA was dissolved in 200 mL of water containing 6 mL of 1 M NaOH at 70°C. The pH of the solution was adjusted to 7.1~7.2 using either HCl or NaOH. The solution was then allowed to cool naturally, with slow stirring at room temperature, followed by overnight storage in an isopropanol bath at 4°C. The resulting precipitate was filtered from the solution and dried under low heat.(Lin et al., 2022).

1.8 Biosafety Profile *in vivo*

Serum levels of alanine aminotransferase (ALT), creatinine (CREA), urea (UREA), and aspartate aminotransferase (AST) were measured 7 and 30 days after administration of Uri@RBC to assess safety. Additionally, major tissues including liver, kidney, spleen, lung, and heart were collected from mice at these time points. Tissues were fixed in 4% formalin, sectioned, and stained with hematoxylin and eosin (H&E) for histological examination.

1.9 Statistical analysis.

Data were presented as mean ± SEM and analyzed using GraphPad Prism 8.0 software. Statistical analysis involved Student's t-test for comparing two groups and one-way ANOVA or 2-way ANOVA for multiple groups. A significance level of $p < 0.05$ was considered statistically significant. ***$p < 0.001$, **$p < 0.01$, *$p < 0.05$.
